# Glucagon-like peptide-1 receptor agonists and diabetic retinopathy: nationwide cohort and Mendelian randomization studies

**DOI:** 10.1186/s12916-023-02753-6

**Published:** 2023-02-03

**Authors:** Deqiang Zheng, Ning Li, Rui Hou, Xiaoyu Zhang, Lijuan Wu, Jan Sundquist, Kristina Sundquist, Jianguang Ji

**Affiliations:** 1 Department of Epidemiology and Health Statistics, School of Public Health, Capital Medical University, Beijing, China; 2 Department of Clinical Sciences Malmö, Center for Primary Health Care Research, Lund University, Lund, Sweden; 3 Department of Anesthesiology, Sanbo Brain Hospital, Capital Medical University, Beijing, China; 4 Department of Family Medicine and Community Health, Department of Population Health Science and Policy, Icahn School of Medicine at Mount Sinai, New York, USA; 5 Department of Functional Pathology, School of Medicine, Center for Community-based Healthcare Research and Education (CoHRE), Shimane University, Matsue, Japan

**Keywords:** GLP-1 RAs, Diabetic retinopathy, Cohort study, Mendelian randomization

## Abstract

**Background:**

The ability of glucagon-like peptide-1 receptor agonists (GLP-1 RAs) to decrease certain microvascular events has called for the investigation of GLP-1 RAs against diabetic retinopathy (DR), but the evidence is limited. By combining data from observational and Mendelian randomization (MR) studies, we aimed to investigate whether GLP-1 RAs decrease the risk of DR.

**Methods:**

We combined data from several Swedish Registers and identified patients with incident type 2 diabetes being treated with GLP-1 RAs between 2006 and 2015, and matched them to diabetic patients who did not use GLP-1 RAs as the comparisons. The Cox proportional hazards models were applied to assess the risk of DR. We further performed the summary-data-based MR (SMR) analyses based on the Genotype-Tissue Expression databases and the Genome-Wide Association Study of DR from the FinnGen consortium.

**Results:**

A total of 2390 diabetic patients were treated with GLP-1 RAs and the incidence of DR was 5.97 per 1000 person-years. Compared with diabetic patients who did not use GLP-1 RAs having an incidence of 12.85 per 1000 person-years, the adjusted hazard ratio (HR) of DR was 0.42 [95% confidence interval (CI), 0.29–0.61]. Genetically-predicted *GLP1R* expression (the target of GLP-1 RAs) showed an inverse association with background [odds ratio (OR)=0.83, 95% CI, 0.71–0.97] and severe nonproliferative DR (OR=0.72, 95% CI, 0.53–0.98), and a non-significant association with overall (OR=0.97, 95% CI, 0.92–1.03) and proliferative DR (OR=0.98, 95% CI, 0.91–1.05).

**Conclusions:**

Both observational and mendelian randomization analyses showed a significantly lower risk of DR for patients treated with GLP-1 RAs, which calls for further studies to validate these findings.

**Supplementary Information:**

The online version contains supplementary material available at 10.1186/s12916-023-02753-6.

## Background

Diabetic retinopathy (DR) is a common complication of diabetes mellitus [1], which in 2020 affected more than 100 million individuals worldwide. By 2045 it is expected that about 160 million people could be affected by DR [2]. The cumulative incidence of DR among patients with diabetes was relatively high, although the treatment strategies including pharmacological and non-pharmacological approaches against diabetes have evolved greatly during recent decades. DR can be classified as background DR, severe nonproliferative DR, and proliferative DR based on its severity. Specifically, microaneurysms can be found in the fundus for the stage of background DR; severe nonproliferative DR includes intraretinal hemorrhages, venous beading, and moderate or prominent intraretinal microvascular abnormalities; proliferative DR includes vitreous and hemorrhages or neovascularization [3]. It is thus highly important to find new approaches to prevent DR development or arrest its progression.

Glucagon-like peptide-1 receptor agonists (GLP-1 RAs) have been shown to reduce glycated hemoglobin levels via regulation of incretin function without secondary effects such as weight gain and hypoglycemia [4, 5]. Based on the evidence from multi-center long-term cardiovascular outcome trials (CVOTs) which showed that GLP-1 RAs could reduce cardiovascular mortality and the incidence of non-fatal myocardial infarction or non-fatal stroke, GLP-1 RAs have been incorporated into clinical guidelines by the American Diabetes Association (ADA) and the European Association for the Study of Diabetes (EASD) [6–10].

Growing evidence suggests that retinal neurodegeneration is an early event in the pathogenesis of DR [11]. Additionally, *GLP1R* can produce neuroprotective effects in the nervous systems [12]. Furthermore, the expression of *GLP1R* is abundant in the human retina [13]. Therefore, it is reasonable to hypothesize that GLP-1 RAs could prevent or arrest the development of DR. However, the evidence is still limited and inconsistent [14–17]. In a recent cohort study including 444 participants taking GLP-1 RAs, Antonios Douros et al. [16] reported that GLP-1 RAs manifested their protective role of DR when compared with insulin, but the association may be due to residual confounding. A small sample study with 47 patients [18] suggested that GLP-1 RAs can improve the prognosis of DR. However, the REWIND [8] and LEADER [10] trials reported null findings between GLP-1 RAs and DR. By contrast, the SUSTAIN-6 trial suggested a higher risk of DR compared with the placebo group [9]. These inconsistent results might be due to the different definitions of DR in previous studies. In the REWIND study, DR was defined as photocoagulation, anti-vascular endothelial growth factor therapy, or vitrectomy [8], whereas DR was determined when patients had vitreous hemorrhage, the onset of diabetes-related blindness, and the need for treatment with an intravitreal agent or retinal photocoagulation in the SUSTAIN-6 [9] and LEADER trials [10]. Some previous studies had smaller sample sizes and short follow-up times, which cast doubt on the beneficial effects of GLP-1 RAs against DR. Therefore, it is urgent to clarify further whether GLP-1 RAs indeed exert a beneficial effect on DR using a novel study design or study with a large sample size.

In this study, we first accessed the nationwide Swedish Patient Register and identified all the patients diagnosed with incident diabetes. We then combined them with data derived from several nationwide Swedish registers to investigate the subsequent incidence of DR among patients who had ever previously used GLP-1 RAs. Lastly, we then compared them to patients that did not use GLP-1 RAs. Additionally, we adopted another study design, the Mendelian randomization (MR) analysis, to provide more reliable insights into causal associations of GLP-1 RAs with different severity of DR [19, 20].

## Methods

### Study data

The population-based cohort study was done by the linkage of several national Swedish registries. All the patients who were diagnosed with diabetes in Sweden were identified from the Swedish Patient Register, which was launched in 1964 and has had complete nationwide coverage since 1987. From 2001 onwards, outpatient visits have also been included in the register. The admission and discharge dates, as well as the main discharge diagnosis and secondary diagnoses, are included in each record [21]. These patients were further linked to the Swedish Prescribed Drug Registry to retrieve their medication records. The Prescribed Drug Registry was established on July 1, 2005, and contained all prescriptions for medications dispensed by Swedish pharmacies to the entire Swedish population [22]. Patients were included if they met all the following criteria: (1) new-onset type 2 diabetes diagnosed between 1 January 2006 and 31 December 2015, according to International Classification of Diseases, Revision 10 (ICD-10) code (Additional file 1: Table S1) or use of antidiabetic medications by the Anatomical Therapeutic Chemical (ATC) classification codes (Additional file 1: Table S2); (2) being treated with metformin, sulfonylureas or GLP-1 RAs during follow up; (3) without a history of other types of diabetes, such as type 1 diabetes or gestational diabetes (Additional file 1: Table S3).

**Fig. 1:**
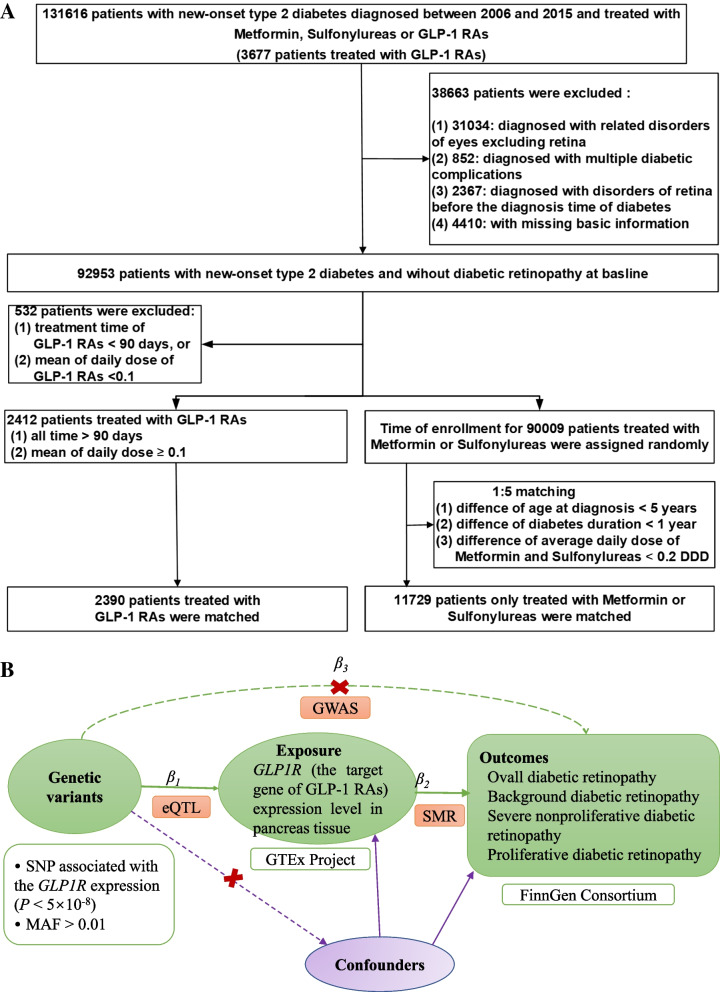
Identification of eligible patients and development of cohorts in the study and the design of summary-data-based Mendelian randomization (SMR) model. **A** Identification of eligible patients and development of cohorts in the observational study. GLP-1 RAs, Glucagon-like peptide-1 receptor agonists; DDD, Defined daily dose. **B** The design of summary-data-based Mendelian randomization (SMR) model. **×**: there are no associations between genetic variants and exposure (or outcome). GWAS, Genome-Wide Association Study; eQTL, Expression quantitative trait loci; SMR, Summary-data-based Mendelian randomization; SNP, Single nucleotide polymorphism; MAF, Minor allele frequency

We then excluded patients that met any of the following criteria: (1) diagnosis with related disorders of the eyes excluding the retina based on the ICD-9 and ICD-10 codes (Additional file 1: Table S4); (2) diagnosis with multiple diabetic complications (E11.7 by the ICD-10); (3) diagnosis with disorders of the retina before the diagnosis of type 2 diabetes (Additional file 1: Table S5); (4) with missing information of the diagnosis date, education level, and birth country; (5) treatment duration of GLP-1 RAs < 90 days or an average daily dose of GLP-1 RAs < 0.1 (unit: defined daily dose, DDD) during the follow-up; (6) treatment with insulin. The flowchart of the study population is presented in Fig. 1A.

Summary-data-based Mendelian randomization (SMR) method is a novel MR approach integrating cis-expression quantitative trait loci (cis-eQTL) and the summary-level data from Genome-Wide Association Study (GWAS) to estimate the effects of gene expression levels on outcomes. The cis-eQTL genetic variants were used as the instrumental variable for gene expression of *GLP1R,* the target gene of GLP-1 RAs. The SMR approach is shown in Fig. 1B. We used the eQTL summary data from Genotype-Tissue Expression (GTEx) project (https://gtexportal.org/) [23–25]. The GWASs for DR (18,097 cases, 206,364 controls) [26] and different severity of DR, including background DR (2510 cases, 242,308 controls) [27], severe nonproliferative DR (568 cases, 242,308 controls) [28] and proliferative DR (10,860 cases, 242,308 controls) [29] were received from the FinnGen consortium (https://r6.finngen.fi/). The details of the data sets are presented in Additional file 1: Table S6.

### Exposure and follow-up

The first dispensing date of GLP-1 RAs (including liraglutide, semaglutide, and dulaglutide) was served as the time of cohort entry in the GLP-1 RAs group. Subsequently, each patient in the non-GLP-1 RAs group was randomly assigned a date as the time point of enrollment (Additional file 1: Fig. S1). Each patient prescribed with GLP-1 RAs was matched with up to five comparisons — who did not use GLP-1 RAs — conditional on the same sex, the age at the diagnosis, the duration of diabetes before enrolling in the cohort study, and the average dose of metformin and sulfonylureas before the administration of GLP-1 RAs (Additional file 1: Fig. S1). Medication was identified according to the ATC classification codes. Participants were followed up from cohort entry to the occurrence of DR, death from any cause, or 31 December 2018, whichever occurred first.

### Outcome and covariates

Diabetic retinopathy was identified by using the ICD-10 code (H36.0 or E11.3). The information on DR diagnosis was extracted from the Swedish Patient Register. Death was considered a censored event. A group of possible confounders was adjusted in this study including the age at diagnosis of diabetes, sex, birth country, education level, duration of diabetes, the history of hypertension and cardiovascular disease (CVD) (Additional file 1: Table S7), and average daily dosage of antidiabetic drugs (metformin, sulfonylureas or GLP-1 RAs) after cohort entry. Education level was classified into three groups: <= 9 years, 10 to 12 years, and >12 years.

In the SMR analysis, the primary outcomes were DR and different severity of DR. Specifically, background DR (ICD 10: H36.00), severe nonproliferative DR (ICD 10: H36.02), and proliferative DR (ICD 10: H36.03) were identified by using the ICD codes.

### Statistical analyses

To describe the characteristics of patients in the cohort study, mean and standard deviation (SD) were presented for continuous data, and number (*n*) and percentage (%) were presented for categorical covariates. Covariate balance after matching was assessed using standardized differences. The standardized differences of 10% or less indicated appropriate matching. We estimated separately the incidence rate of DR for the GLP-1 RAs group and non-GLP-1 RAs group. Cumulative incidence was estimated with the Kaplan-Meier method and the log-rank test was performed to compare the difference. The adjusted Cox proportional hazards models were applied to assess the association of GLP-1 RAs with the risk of DR. Hazard ratio (HR) and corresponding 95% confidence interval (CI) were calculated from adjusted Cox regression. Four models were established for the statistical analyses in this study. Model 1 was a crude analysis without any adjustment, and model 2 was adjusted for sex, age at diagnosis of diabetes, education level, and birth country. Model 3 was adjusted for the variables in model 2, as well as for the duration of diabetes and the history of hypertension and CVD. Model 4 was adjusted for variables in model 3, as well as for the average daily dosage of metformin and sulfonylureas after the cohort entry, and other retinal disorders.

### Sensitivity analyses of the observational study

We used four sensitivity analyses to validate the consistency of our results. First, we assessed the robustness of the HR in a multivariable Cox model by randomly assigning the index date in the non-GLP-1 RAs group five times. We then performed multivariable Cox regressions using these newly randomly identified non-GLP-1 RAs controls as the reference to evaluate the sensitivity of the results. Second, considering the biological latency of GLP-1 RAs as well as the potential underdiagnosis of DR at baseline, we, therefore, undertook the analysis by excluding patients with less than 6 months of follow-up. Third, given that the effect of GLP-1 RAs on DR may differ by sex, we performed subgroup analyses by stratifying the patients into two groups of men and women. Fourth, to assess the influence of unmeasured confounding, the E value was calculated based on the estimated HR for DR [30].

### SMR analyses

For the SMR analyses, we identified single nucleotide polymorphisms (SNPs) significantly associated (*P* < 5×10^−8^) with the expression of the *GLP1R* gene in pancreatic tissue (based on the fact that GLP-1 RAs control glucose homeostasis by regulating the secretion of insulin and glucagon through *GLP1R* in the pancreas) and filtered for minor allele frequency (MAF) >0.01 to proxy the administration of GLP-1 RAs. Subsequently, we used SMR software to perform allele harmonization between exposure and outcomes. Finally, the odds ratios (ORs) and 95% CIs were calculated to provide evidence for an underlying causal association of expression of the *GLP1R* gene with the risk of DR and different severity of DR. The strength of the genetic instrument was evaluated using the F-statistic, and SNPs with F-statistic >10 were included to avoid weak instrument bias. The heterogeneity in dependent instruments (HEIDI) test (*P* < 0.01) was leveraged to distinguish pleiotropy from the linkage.

A significance level of *P* < 0.05 was considered in all analyses. Statistical analyses were performed in R (version 4.0.1), SAS (version 9.4), and SMR software, version 1.03 (https://yanglab.westlake.edu.cn/software/smr/#Download).

### Role of the funding source

The funders of the study had no role in study design, data collection, data analysis, data interpretation, or writing of the report.

## Results

In the primary analysis, we included a total of 14,119 patients, including 2390 patients who were prescribed GLP-1 RAs and 11,729 randomly selected patients without administration of GLP-1 RAs. The average age at the diagnosis of diabetes and the mean diabetes duration were 53.19±10.38 years and 4.19±2.77 years, respectively. The baseline covariates were well balanced (standardized difference < 10%) after matching (Table 1). The median follow-up time was 2.03 (interquartile range: 1.07–3.18) years for the GLP-1 RAs group and 1.92 (interquartile range: 0.99–3.47) years for the non-GLP-1 RAs group (Table 1).

**Table 1:** Characteristics of the study population

	GLP-1 RAs (***n*** = 2390)	Non-GLP-1 RAs (***n*** = 11,729)	Standardized difference, %^**a**^
**Matched variables**			
Age at diabetes diagnosis (years)	52.65 (10.38)	53.30 (10.37)	6.29
Sex, *n* (%)			0.82
Men	1315 (55.02)	6501 (55.43)	
Women	1075 (44.98)	5228 (44.57)	
Duration of diabetes (years)	4.22 (2.82)	4.18 (2.76)	1.31
Average daily dose before cohort entry			
Metformin (DDD)	0.555 (0.296)	0.530 (0.293)	8.45
Sulfonylureas (DDD)	0.0537 (0.143)	0.0442 (0.124)	7.08
**Unmatched variables**			
Educational level, *n* (%)			13.75
<10 years	481 (20.12)	2961 (25.25)	
10–12 years	1236 (51.72)	5438 (46.36)	
>12 years	673 (28.16)	3330 (28.39)	
Country of birth, *n* (%)			30.62
Sweden	1980 (82.85)	8210 (70.00)	
Abroad	410 (17.15)	3519 (30.00)	
Medical history			
Hypertension	1908 (79.83)	8452 (72.06)	18.26
Cardiovascular diseases	370 (15.48)	1741 (14.84)	1.78
Other retinal disorders	7 (0.29)	40 (0.34)	0.86
Average daily dose after cohort entry			
Metformin (DDD)	0.629 (0.372)	0.560 (0.350)	19.08
Sulfonylureas (DDD)	0.0741 (0.220)	0.0831 (0.218)	4.12
GLP-1 RAs (DDD)	0.788 (0.228)	-	-

The data were expressed as mean (standard deviation) for continuous variables and numbers (percentages) for categorical variables. *P*-value: from the Wilcoxon rank-sum test for continuous variables and chi-square test for categorical variables^a^

The absolute value of the difference in means or proportions between the GLP-1 RAs group and the non-GLP-1 RAs control group divided by the pooled SD. Values of 10% or less indicate appropriate matching. *DDD* Defined daily dose

### Primary end point

**Fig. 2:**
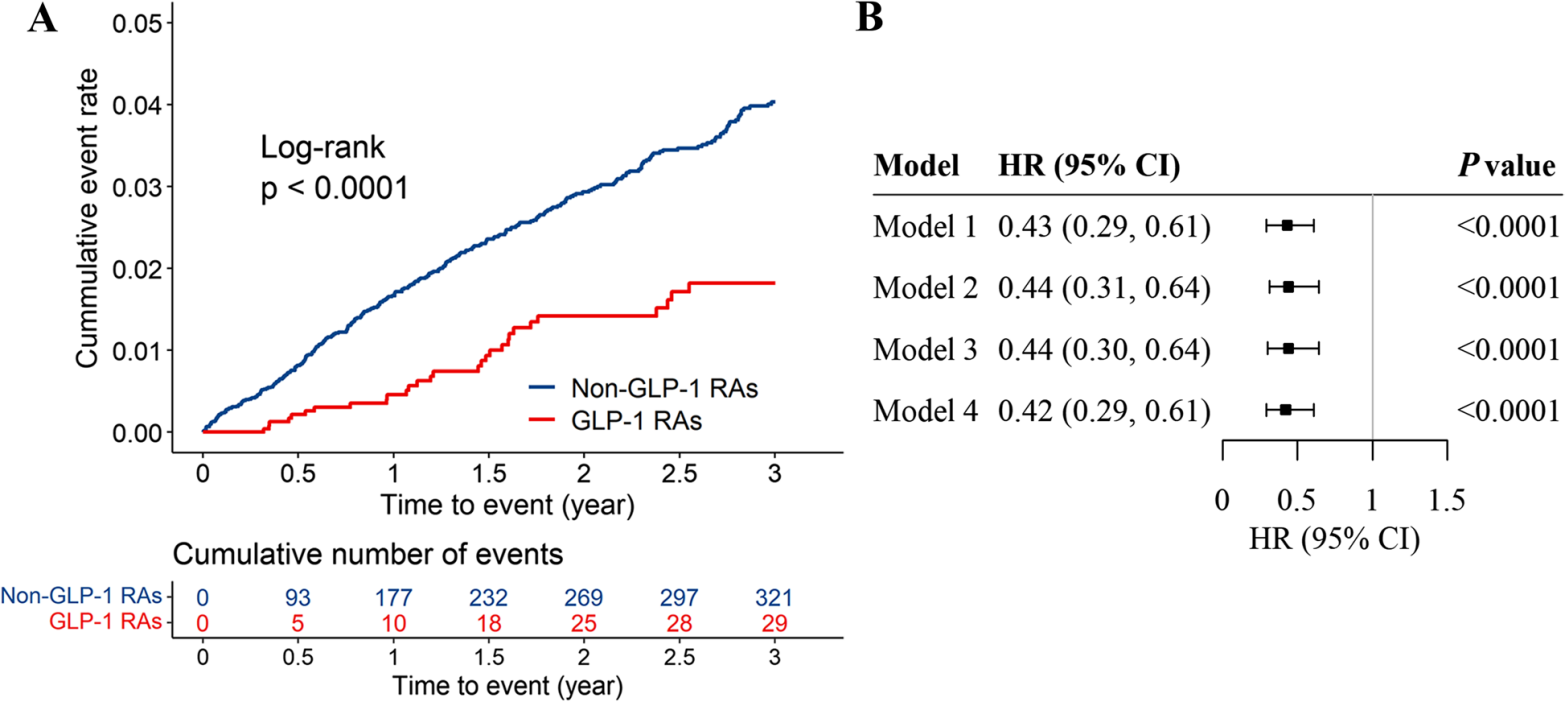
Cumulative incidence estimates (Kaplan-Meier) for DR and the forest plot about the association between GLP-1 RAs and DR using Cox regression. DR, Diabetic retinopathy; GLP-1 RAs, Glucagon-like peptide-1 receptor agonists. **A** Cumulative incidence estimates (Kaplan-Meier) for DR in non-GLP-1 RAs and GLP-1 RAs groups. The blue and red lines indicate the cumulative incidence rate of the non-GLP-1 RAs group and the GLP-1 RAs group, separately. DR, Diabetic retinopathy; GLP-1 RAs, Glucagon-like peptide-1 receptor agonists. **B** The forest plot about the association between GLP-1 RAs and DR using Cox regression for four models. The association between GLP-1 RAs and DR was significant in all models. HR, Hazard ratio; CI, Confidence interval; GLP-1 RAs, Glucagon-like peptide-1 receptor agonists; DR, Diabetic retinopathy

During follow-up, 31 new incident cases of DR were identified in the GLP-1 RAs group and 398 in the non-GLP-1 RAs group. The incidence rate of DR was 5.97 events per 1000 person-years for GLP-1 RAs users and 12.85 events per 1000 person-years for non-GLP-1 RAs users. The cumulative incidence of DR was significantly different between the two groups (Fig. 2A). In the unadjusted model, the GLP-1 RAs users showed a lower risk for DR compared with nonusers (HR, 0.43; 95% CI, 0.29–0.61; *P*< 0.0001). After further adjustment for potential confounders, model 2 and model 3 had an HR similar to model 1. In the fully adjusted model, compared with non-GLP-1 RAs users, the HR of DR in the patients treated with GLP-1 RAs was 0.42 (95% CI, 0.29–0.61) (Fig. 2B).

### Sensitivity analyses

**Fig. 3:**
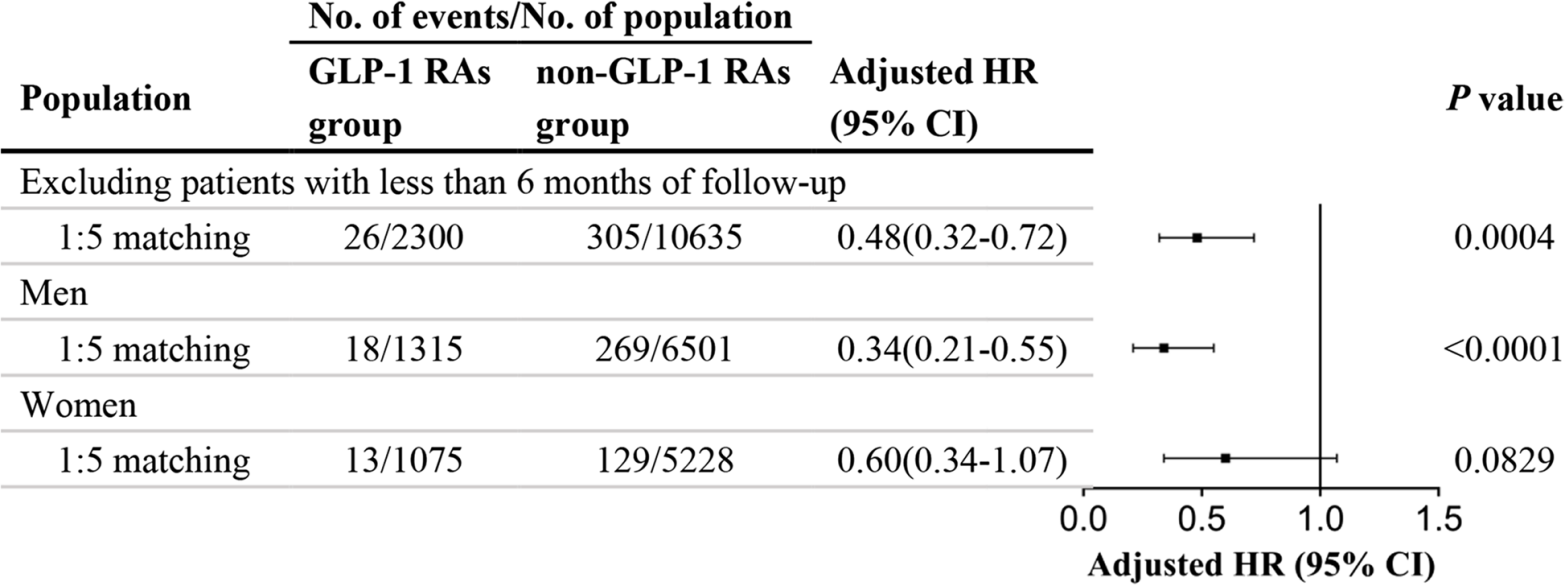
Association of GLP-1 RAs and DR using Cox regression in the sensitivity and subgroup analyses. The treatment of GLP-1 RAs was also associated with the risk of DR after excluding the patients with less than 6 months of follow-up. Subgroup analyses indicated GLP-1 RAs were associated with DR in men but not in women. GLP-1 RAs, Glucagon-like peptide-1 receptor agonists; HR, Hazard ratio; CI, Confidence interval; DR, Diabetic retinopathy

Results of the sensitivity analyses using other randomly selected controls as the references are presented in Additional file 1: Fig. S2 and Additional file 1: Table S8. The administration of GLP-1 RAs reduced the risk of DR in all datasets. The median HR was 0.41 (range 0.39–0.43). After excluding 1184 patients (90 patients in the GLP-1 RAs group and 1094 patients in the non-GLP-1 RAs group) with less than 6 months of follow-up, the HR of DR in the patients treated with GLP-1 RAs was 0.48 (95% CI, 0.32–0.72) compared with non-GLP-1 RAs users in the fully adjusted Cox regression model (Fig. 3).

The association between GLP-1 RAs and the risk of DR was similar in men and women (Fig. 3). Among male patients, 18 new incident cases of DR were identified in the GLP-1 RAs group and 269 cases were identified in the non-GLP-1 RAs group. Male patients taking GLP-1 RAs had a reduced risk of DR in the fully adjusted model (HR, 0.34; 95% CI, 0.21–0.55). Among female patients, 14 and 92 first-ever incident cases of DR were diagnosed in the GLP-1 RAs group and nonusers, respectively. Among women, there was also an inverse association of the administration of GLP-1 RAs with the risk of DR (HR, 0.60; 95% CI, 0.34–1.07), but it was not statistically significant (*P* = 0.0829).

Additionally, the estimated *E* value was 4.19, and the confidence limit was 2.66, suggesting that the observed association between the administration of GLP-1 RAs and DR can only be explained by an unmeasured confounder when the confounding factor could meet the following criteria: confounder–GLP-1 RAs relative risk > 4.19 (or relative risk < 0.24) and confounder–DR relative risk > 4.19 (or relative risk < 0.24).

### SMR analyses

As presented in Table 2 and Additional file 1: Fig. S3, the results from SMR analyses indicated that a 1-SD increase of *GLP1R* gene expression (target for GLP-1 RAs, probe: ENSG00000112164) in pancreas tissue was associated with the decreased risk of background DR (OR, 0.83; 95% CI, 0.71–0.97; *P* = 0.0162) and severe nonproliferative DR (OR, 0.72; 95% CI, 0.53–0.98; *P* = 0.0355). However, the association of *GLP1R* gene expression with overall DR (OR, 0.97; 95% CI, 0.92–1.03; *P* = 0.3471) and proliferative DR (OR, 0.98; 95% CI, 0.91–1.05; P = 0.5256) was not significant. The F-statistic for the selected instrument variant (rs2268650) was 49.25, suggesting that there was little possibility of weak instrument bias in our study. The HEIDI test manifested that the analyses of *GLP1R* with background DR (*P* = 0.29), and severe nonproliferative DR (*P* = 0.42) were not due to linkage.

**Table 2:** The association of a 1-SD increase of *GLP1R* expression in pancreas tissue with the risk of DR

Gene	SNP	A1/A2	eQTL association				GWAS association				SMR association	
			Beta	Se	***P*** value	***F***	Outcome	Beta	Se	***P*** value	OR (95% CI)	***P*** value
*GLP1R*	rs2268650	A/G	−0.42	0.06	2.26×10^−12^	49.25	DR	0.01	0.01	0.34	0.97 (0.92–1.03)	0.3471
*GLP1R*	rs2268650	A/G	−0.42	0.06	2.26×10^−12^	49.25	Background DR	0.08	0.03	0.01	**0.83 (0.71–0.97)**	**0.0162**
*GLP1R*	rs2268650	A/G	−0.42	0.06	2.26×10^−12^	49.25	Severe nonproliferative DR	0.14	0.06	0.03	**0.72 (0.53–0.98)**	**0.0355**
*GLP1R*	rs2268650	A/G	−0.42	0.06	2.26×10^−12^	49.25	Proliferative DR	0.01	0.02	0.52	0.98 (0.91–1.05)	0.5256

*SD* Standard deviation, *DR* Diabetic retinopathy, *SMR* Summary-data-based Mendelian randomization, *SNP* Single nucleotide polymorphism, *GWAS* Genome-Wide Association Study, *eQTL* Expression quantitative trait loci, *A1* effect allele, *A2* other allele, *OR* Odds ratio, *CI* Confidence interval

## Discussion

This study combined evidence from a nationwide population-based cohort study and a study using MR study design to provide more robust causal inferences between the use of GLP-1 RAs and the risk of DR in patients with type 2 diabetes mellitus. From the nationwide prospective cohort study, we found that the administration of GLP-1 RAs was associated with a significantly decreased risk of DR, which showed robustness in different sensitivity analyses. The SMR analyses reported inverse associations of the expression of the *GLP1R* gene with background and severe nonproliferative DR, suggesting that administration of GLP-1 RAs, which proxy an increased expression of the *GLP1R* gene, might causally decrease the risk of DR.

The CVOTs have evaluated the associations between GLP-1 RAs and the risk of DR [8–10, 31]. However, the results were inconsistent. The different definitions of DR in the previous studies possibly resulted in inconsistency. The cohort study, which used the UK Clinical Research Datalink, found that GLP-1 RAs were not related to a decreased risk of DR when compared with two or more antidiabetic drugs [16]. However, there were only 444 patients with the administration of GLP-1 RAs; this might not be powerful enough to detect the significant difference. In addition, the treatment duration among patients taking two or more antidiabetic drugs was shorter than patients taking GLP-1 RAs, which possibly results in a low incidence of DR and a biased association. Conversely, Lakshminarayanan Varadhan et al. [18] suggested that although GLP-1 RAs were related to a transient worsening of DR because of the rapid improvement in glycemic control, 71% of diabetes patients showed improvement of DR after continuing GLP-1 RAs treatment. Our study indicated that taking GLP-1 RAs was associated with a significant reduction in the risk of DR. In addition, we found that the expression of *GLP1R* was associated with background and nonproliferative DR, but not associated with later stages of DR progression (proliferative DR) using MR method. GLP-1 RAs could inhibit the expression of plasminogen activator inhibitor type-1 (PAI-1) and vascular adhesion molecule (VAM) in human vascular endothelial cells in vitro, which had a protective effect against endothelial cell dysfunction (ECD) in the early stages of diabetic vascular disease [32] and might explain the lower incidence of background and severe nonproliferative DR among individuals who used GLP-1 RAs. Bénédicte Gaborit et al. [17] reported that exendin-4 (GLP-1R agonist) did not exert any effect on retinal neovascularization in vivo, which is a key characteristic of proliferative DR. Therefore, the administration of GLP-1 RAs might not affect the risk of proliferative DR. Consistent with this finding, the AngioSafe type 2 diabetes study confirmed no association between GLP-1 RAs and severe DR, but it did not assess the relationship with background DR [17]. As the different severity of DR was not well distinguished in the Swedish Patient Register, evaluating the effect of GLP-1 RAs on various severities of DR was not possible in this cohort study. Our study suggested that using GLP-1 RAs will inhibit the initiation of DR among patients with diabetes, mainly background and nonproliferative DR, which could be recommended to use as a first-line treatment to prevent DR.

The pathogenic mechanisms of onset and progression of DR are not completely clear. Molecular and biochemical mechanisms mainly included the increased oxidative stress through polyol pathway flux, the accumulation of advanced glycation end products, the hexosamine pathway, and the protein kinase C pathway. Subsequently, oxidative stress could result in apoptosis of retinal cells, inflammation, and ultimately DR [33]. Correspondingly, the treatment of GLP-1 RAs for one week in rats with type 2 diabetes could reduce NOX3 and SOD2 levels in the retina cells, alleviate autophagy through the GLP-1R-ERK1/2-HDAC6 signaling pathway, and finally improve DR [34]. Furthermore, there have been prior studies found that the onset of DR was associated with the pathology of the retinal neurovascular unit [35, 36]. Retinal neurodegeneration was an early event in the pathogenesis of DR [11, 37, 38]. GLP-1 RAs exerted neuroprotective effects by inhibiting glutamate accumulation to prevent excitotoxicity and neural apoptosis [39], which possibly explains the protective effects of the *GLP1R* gene on background DR and severe nonproliferative DR, but not proliferative DR.

In comparison with previous studies, this study has several strengths. First, in conjunction with combining data derived from several Swedish national registers, we obtained accurate information on prescription and the diagnosis of DR, as well as the other factors possibly related to the development and progression of DR. Second, this was the first study with a longer follow-up period, longer duration of diabetes and large sample size to investigate the association between GLP-1 RAs and DR using real-world clinical data. Third, the matching methods adopted in this study mimicked a randomized controlled trial, which provides a higher level of evidence. For example, performing matching could provide greater comparability between the GLP-1 RAs users and nonusers. In addition, the baseline covariates after matching were fully balanced. Fourth, multiple sensitivity analyses were conducted to validate the robustness of the results. Finally, we obtained the consistent results of the association of GLP-1 RAs and DR thoroughly by incorporating information from an observational study and an MR analysis. However, there were several limitations in our study. First, the information on the severity of DR was not available in the Swedish Patient Register. However, we performed the SMR analyses to explore the association between GLP1R expression and different severity of DR. Second, the misclassification of DR from the Swedish Patient Register can not be fully excluded. However, the positive predictive value of the diagnosis in the Swedish Patient Register was generally very high (ranging between 85 and 95%) based on the previous report [40]. Third, the study population focused on European ancestry, which might limit the generalization of our findings to other populations. Fourth, we could not adjust the effect of glycosylated hemoglobin A1c and glycemic control in our population-based study due to the lacking of this information in our database. However, we argue that lacking adjustment of these factors will not bias our results because these factors might act as mediators instead of confounding factors based on the available evidence that poor glucose control will lead to DR, whereas using GLP-1 RAs will help to control glucose control. Additionally, the estimated HR should be higher if the glycosylated hemoglobin A1c and glycemic control are the confounding factors in this study because individuals who used GLP-1 RAs (as second-line treatment for diabetes in Sweden) in our study could have poor glucose control compared to those individuals who did not use GLP-1 RAs. Besides, our MR analyses, which are independent of reverse causality and residual confounding, showed a similar result, further supporting our observation. Finally, residual confounding could not be fully excluded in our population-based analyses although we have tried to adjust a couple of demographic and clinical factors. However, the E value calculation showed that to explain away the observed associations of GLP-1 RAs use and DR, an unmeasured confounder would need to have an HR of 4.19 with both GLP-1 RAs use and DR. Therefore, weaker confounding could not account for the observed association. The E value indicated the robustness of the observed association in our study.

## Conclusions

In conclusion, we found a lower risk of DR among patients with diabetes who had ever previously used GLP-1 RAs from real-world clinical data as well as genetically predicted administration of GLP-1 RAs. Although our findings may support the use of GLP-1 RAs as first-line type 2 diabetes treatment for the prevention of DR, further research such as a randomized clinical trial study with a longer follow-up time is warranted to establish more robust evidence.

## Supplementary Information

**Additional file 1: Table S1.** Diagnosis codes to assist in identifying type 2 diabetes. **Table S2.** Prescription codes to assist in identifying anti-diabetic drugs. **Table S3.** Diagnosis codes to assist in identifying other types of diabetes excluding type 2 diabetes. **Table S4.** Diagnosis codes to assist in identifying pre-existing conditions that could disqualify patients for study eligibility. **Table S5.** Diagnosis codes to assist in identifying retinal disorders. **Table S6.** Detailed information of eQTL and GWAS summary data in SMR analyses. **Table S7.** Diagnosis codes to assist in identifying baseline hypertension and CVD. **Table S8.** Hazard ratios and 95% confidence intervals for the GLP-1 RAs group versus nonusers from fully-adjusted Cox models for DR for five iterations of index date random sampling. **Figure S1.** Matched cohort design. **Figure S2.** Hazard ratios and 95% confidence intervals for the GLP-1 RAs group versus nonusers from fully-adjusted Cox models for DR for five iterations of index date random sampling. **Figure S3.** The association of a 1-SD increase of GLP1R gene expression in pancreas tissue with the risk of DR using SMR method.

## Data Availability

The data used in the observational study are available from the corresponding author upon reasonable request. The summary-level data of Genome-Wide Association Studies (GWASs) used in Mendelian randomization study can be accessed by the FinnGen (http://r6.finngen.fi/) and GTEx (https://gtexportal.org/home/).

## Abbreviations

GLP-1 RAs: Glucagon-like peptide-1 receptor agonists
DR: Diabetic retinopathy
MR: Mendelian randomization
HR: Hazard ratio
SMR: Summary-data-based Mendelian randomization
OR: Odds ratio
CI: Confidence interval
CVOTs: Cardiovascular outcome trials
ICD-10: International Classification of Diseases, Revision 10
ATC: Anatomical Therapeutic Chemical
DDD: Defined daily dose
cis-eQTL: Cis-expression quantitative trait loci
GWAS: Genome-Wide Association Study
CVD: Cardiovascular disease
SD: Standard deviation
SNP: Single nucleotide polymorphism
MAF: Minor allele frequency
HEIDI: Heterogeneity in dependent instruments
