# Supplementary material for: Glucagon-like peptide-1 receptor agonists and diabetic retinopathy: nationwide cohort and Mendelian randomization studies
Source: BMC Med. 2023 Feb 3;21:40. doi: 10.1186/s12916-023-02753-6 (PMC9898966; doi:10.1186/s12916-023-02753-6)
Supplement: Supplementary file 1 — Additional file 1: Table S1. Diagnosis codes to assist in identifying type 2 diabetes. Table S2. Prescription codes to assist in identifying anti-diabetic drugs. Table S3. Diagnosis codes to assist in identifying other types of diabetes excluding type 2 diabetes. Table S4. Diagnosis codes to assist in identifying pre-existing conditions that could disqualify patients for study eligibility. Table S5. Diagnosis codes to assist in identifying retinal disorders. Table S6. Detailed information of eQTL and GWAS summary data in SMR analyses. Table S7. Diagnosis codes to assist in identifying baseline hypertension and CVD. Table S8. Hazard ratios and 95% confidence intervals for the GLP-1 RAs group versus nonusers from fully-adjusted Cox models for DR for five iterations of index date random sampling. Figure S1. Matched cohort design. Figure S2. Hazard ratios and 95% confidence intervals for the GLP-1 RAs group versus nonusers from fully-adjusted Cox models for DR for five iterations of index date random sampling. Figure S3. The association of a 1-SD increase of GLP1R gene expression in pancreas tissue with the risk of DR using SMR method. [file 12916_2023_2753_MOESM1_ESM.docx]

**Additional file**

[Table S1. Diagnosis codes to assist in identifying type 2 diabetes. 2](#_Toc119958454)

[Table S2. Prescription codes to assist in identifying anti-diabetic drugs. 3](#_Toc119958455)

[Table S3. Diagnosis codes to assist in identifying other types of diabetes excluding type 2 diabetes. 4](#_Toc119958456)

[Table S4. Diagnosis codes to assist in identifying pre-existing conditions that could disqualify patients for study eligibility. 5](#_Toc119958457)

[Table S5. Diagnosis codes to assist in identifying retinal disorders. 7](#_Toc119958458)

[Table S6. Detailed information of eQTL and GWAS summary data in SMR analyses. 8](#_Toc119958459)

[Table S7. Diagnosis codes to assist in identifying baseline hypertension and CVD. 9](#_Toc119958460)

[Table S8. Hazard ratios and 95% confidence intervals for the GLP-1 RAs group versus nonusers from fully-adjusted Cox models for DR for five iterations of index date random sampling. 10](#_Toc119958461)

[Figure S1. Matched cohort design. 11](#_Toc119958462)

[Figure S2. Hazard ratios and 95% confidence intervals for the GLP-1 RAs group versus nonusers from fully-adjusted Cox models for DR for five iterations of index date random sampling. 12](#_Toc119958463)

[Figure S3. The association of a 1-SD increase of GLP1R gene expression in pancreas tissue with the risk of DR using SMR method. 13](#_Toc119958464)

# Table S1. Diagnosis codes to assist in identifying type 2 diabetes.

| **Diagnosis** | **Codes** |
| --- | --- |
| Non-insulin-dependent diabetes mellitus | ICD-10: E11 |
| With coma | ICD-10: E11.0 |
| With ketoacidosis | ICD-10: E11.1 |
| With renal complications | ICD-10: E11.2 |
| With ophthalmic complications | ICD-10: E11.3 |
| With neurological complications | ICD-10: E11.4 |
| With peripheral circulatory complications | ICD-10: E11.5 |
| With other specified complications | ICD-10: E11.6 |
| With multiple complications | ICD-10: E11.7 |
| With unspecified complications | ICD-10: E11.8 |
| Without complications | ICD-10: E11.9 |

# Table S2. Prescription codes to assist in identifying anti-diabetic drugs.

| **Types** | **ATC codes** |
| --- | --- |
| Insulins and analogues | A10A |
| Metformin | A10BA02 |
| Sulfonylureas | A10BB |
| Sulfonamides (heterocyclic) | A10BC |
| Combinations of oral blood glucose lowering drugs | A10BD |
| Alpha glucosidase inhibitors | A10BF |
| Thiazolidinediones | A10BG |
| Dipeptidyl peptidase 4 (DPP-4) inhibitors | A10BH |
| Glucagon-like peptide-1 (GLP-1) analogues | A10BJ |
| Sodium-glucose co-transporter 2 (SGLT2) inhibitors | A10BK |
| Other blood glucose lowering drugs, excl. insulins | A10BX |

# Table S3. Diagnosis codes to assist in identifying other types of diabetes excluding type 2 diabetes.

| **Diagnosis** | **Codes** |
| --- | --- |
| Insulin-dependent diabetes mellitus | **ICD-9:** 250.01, 250.03, 250.11, 250.13, 250.21, 250.23, 250.31, 250.33, 250.41, 250.43, 250.51, 250.53, 250.61, 250.63, 250.71, 250.73, 250.81, 250.83, 250.91, 250.93  **ICD-10:** E10 |
| Malnutrition-related diabetes mellitus | **ICD-10:** E12 |
| Other specified diabetes mellitus | **ICD-10:** E13 |
| Unspecified diabetes mellitus | **ICD-9:** 250.09, 250.19, 250.29, 250.39, 250.49, 250.59, 250.69, 250.79, 250.89, 250.99  **ICD-10:** E14 |

# Table S4. Diagnosis codes to assist in identifying pre-existing conditions that could disqualify patients for study eligibility.

| **Diagnosis** | **Codes** |
| --- | --- |
| Agents primarily affecting skin and mucous membrane, ophthalmological, otorhinolaryngological and dental drugs-ophthalmological drugs and preparations | **ICD-9**: E946.5  **ICD-10**: Y56.5 |
| Burn and corrosion confined to eye and adnexa | **ICD-9**: 940  **ICD-10**: T26 |
| Carcinoma in situ of other and unspecified sites-eye | **ICD-9**: 234.0  **ICD-10**: D09.2 |
| Carcinoma in situ of other and unspecified sites-eye | **ICD-9**: 232.1  **ICD-10**: D04.1 |
| Congenital anomalies of eye | **ICD-9**: 743  **ICD-10**: Q10, Q11, Q12, Q13, Q14, Q15 |
| Disorders of cornea | **ICD-9**: 370, 371  **ICD-10**: H16, H17, H18, H19 |
| Disorders of lens | **ICD-9**: 366  **ICD-10**: H25-H28 |
| Disorders of optic nerve and visual pathways | **ICD-9**: 377  **ICD-10**: H46-H48 |
| Injury of eye and orbit | **ICD-9**: 870, 871,  918, 921  **ICD-10**: S05 |
| Injury to optic nerve and pathways | **ICD-9**: 950  **ICD-10**: S04.0 |
| Malignant melanoma of skin- Eyelid, including canthus | **ICD-9**: 172.1  **ICD-10**: C43.1 |
| Malignant neoplasm of eye | **ICD-9**: 190  **ICD-10**: C69 |
| Malignant neoplasm of spinal cord, cranial nerves and other parts of central nervous system- Optic nerve | **ICD-10**: C72.3 |
| Melanoma in situ of eyelid, including canthus | **ICD-10**: D03.1 |
| Neuromyelitis optica [Devic] | **ICD-9**: 341.0  **ICD-10**: G36.0 |
| Other disorders of choroid | **ICD-9**: 363.4, 363.5, 363.6, 363.7, 363.8, 363.9  **ICD-10**: H31 |
| Other malignant neoplasm of skin-Eyelid, including canthus | **ICD-9**: 173.1 |

**Table S4.** Diagnosis codes to assist in identifying pre-existing conditions that could disqualify patients for study eligibility (continued).

| **Diagnosis** | **Codes** |
| --- | --- |
| Other disorders of eye and adnexa | **ICD-9**: 379  **ICD-10**: H55-H59 |
| Personal history of malignant neoplasm of other sites-eye | **ICD-9**: V10.84  **ICD-10**: Z85.8 |
| Poisoning by topical agents primarily affecting skin and mucous membrane and by ophthalmological, otorhinolarygological and dental drugs - ophthalmological drugs and preparations | **ICD-9**: 976.5  **ICD-10**: T49.5 |
| Visual disturbances and blindness | **ICD-9**: 368, 369  **ICD-10**: H53, H54 |

# Table S5. Diagnosis codes to assist in identifying retinal disorders.

| **Diagnosis** | **Codes** |
| --- | --- |
| Chorioretinal disorders in diseases classified elsewhere | ICD-10: H32 |
| Chorioretinal inflammation | ICD-10: H30 |
| Non-insulin-dependent diabetes mellitus with ophthalmic complications | ICD-10: E11.3 |
| Other retinal disorders | ICD-10: H35 |
| Retinal detachments and breaks | ICD-10: H33 |
| Retinal disorders in diseases classified elsewhere | ICD-10: H36 |
| Retinal vascular occlusions | ICD-10: H34 |

# Table S6. Detailed Information of eQTL and GWAS summary data in SMR analyses.

| **Characteristic** | **Resource** | **Sample size** | **Population ancestry** | **Data download** |
| --- | --- | --- | --- | --- |
| **eQTL data** |  |  |  |  |
| eQTL for *GLP1R* | GTExV8 | Pancreas: 328 | Predominantly European | https://www.gtexportal.org/home/datasets |
| **GWAS summary data** |  |  |  |  |
| Overall DR | FinnGen | 18,097 cases 206,364 controls | European | https://r6.finngen.fi/ |
| Background DR | FinnGen | 2,510 cases 242,308 controls | European | https://r6.finngen.fi/ |
| Severe nonproliferative DR | FinnGen | 568 cases 242,308 controls | European | https://r6.finngen.fi/ |
| Proliferative DR | FinnGen | 10,860 cases 242,308 controls | European | https://r6.finngen.fi/ |

eQTL, expression quantitative trait loci; GWAS, Genome-Wide Association Study; SMR, summary-data-based Mendelian randomization; DR, diabetic retinopathy.

# Table S7. Diagnosis codes to assist in identifying baseline hypertension and CVD.

| **Diagnosis** | **Codes** |
| --- | --- |
| Cerebrovascular diseases | **ICD-9**: 430-438  **ICD-10**: I60-I69 |
| Heart failure | **ICD-9**: 428  **ICD-10**: I50 |
| Hypertensive diseases | **ICD-9**: 401-405  **ICD-10**: I10-I15  **ATC**: C02, C03, C07, C08, C09 |
| Ischemic heart diseases | **ICD-9**: 410-414  **ICD-10**: I20- I25 |

# Table S8. Hazard ratios and 95% confidence intervals for the GLP-1 RAs group versus nonusers from fully-adjusted Cox models for DR for five iterations of index date random sampling.

| **Iteration** | **Hazard ratio** | **95% confidence interval** | ***P* value** |
| --- | --- | --- | --- |
| 1 | 0.418 | 0.289-0.606 | <0.0001 |
| 2 | 0.422 | 0.292-0.611 | <0.0001 |
| 3 | 0.394 | 0.273-0.570 | <0.0001 |
| 4 | 0.407 | 0.281-0.589 | <0.0001 |
| 5 | 0.429 | 0.297-0.622 | <0.0001 |


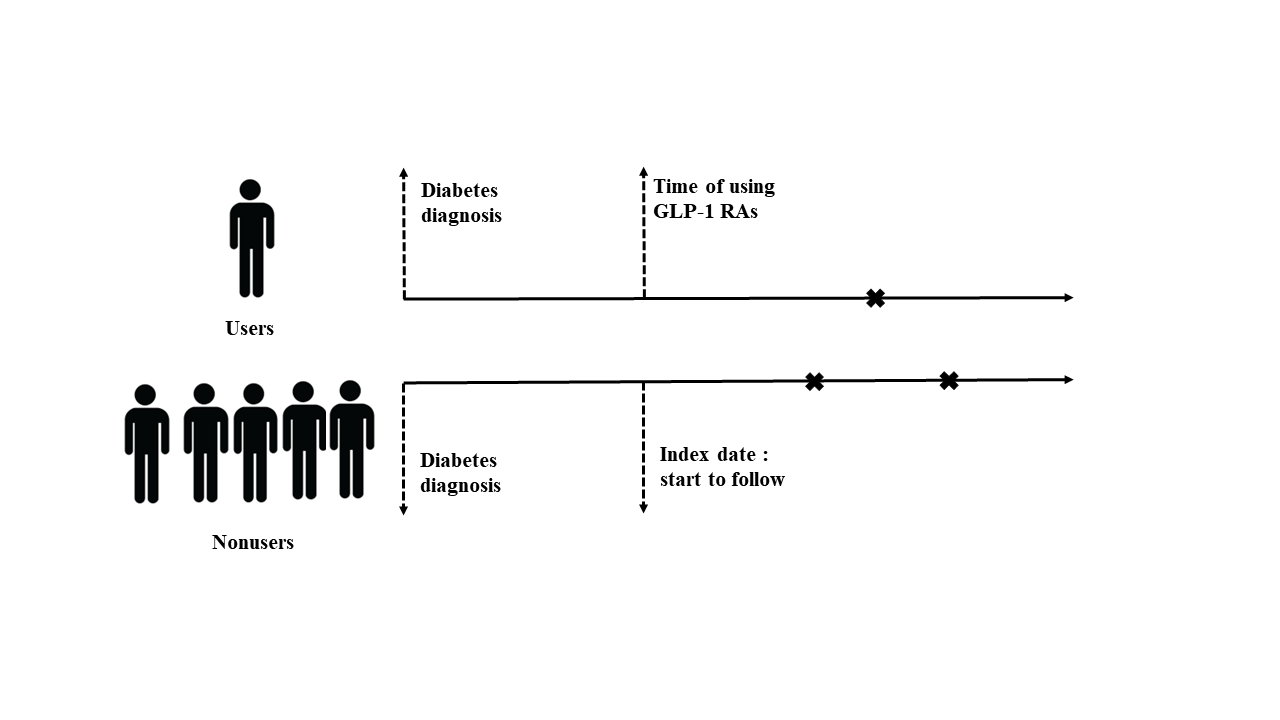


# Figure S1. Matched cohort design.

✖ The time of onset of diabetic retinopathy or death. GLP-1 RAs, glucagon-like peptide-1 receptor agonists.


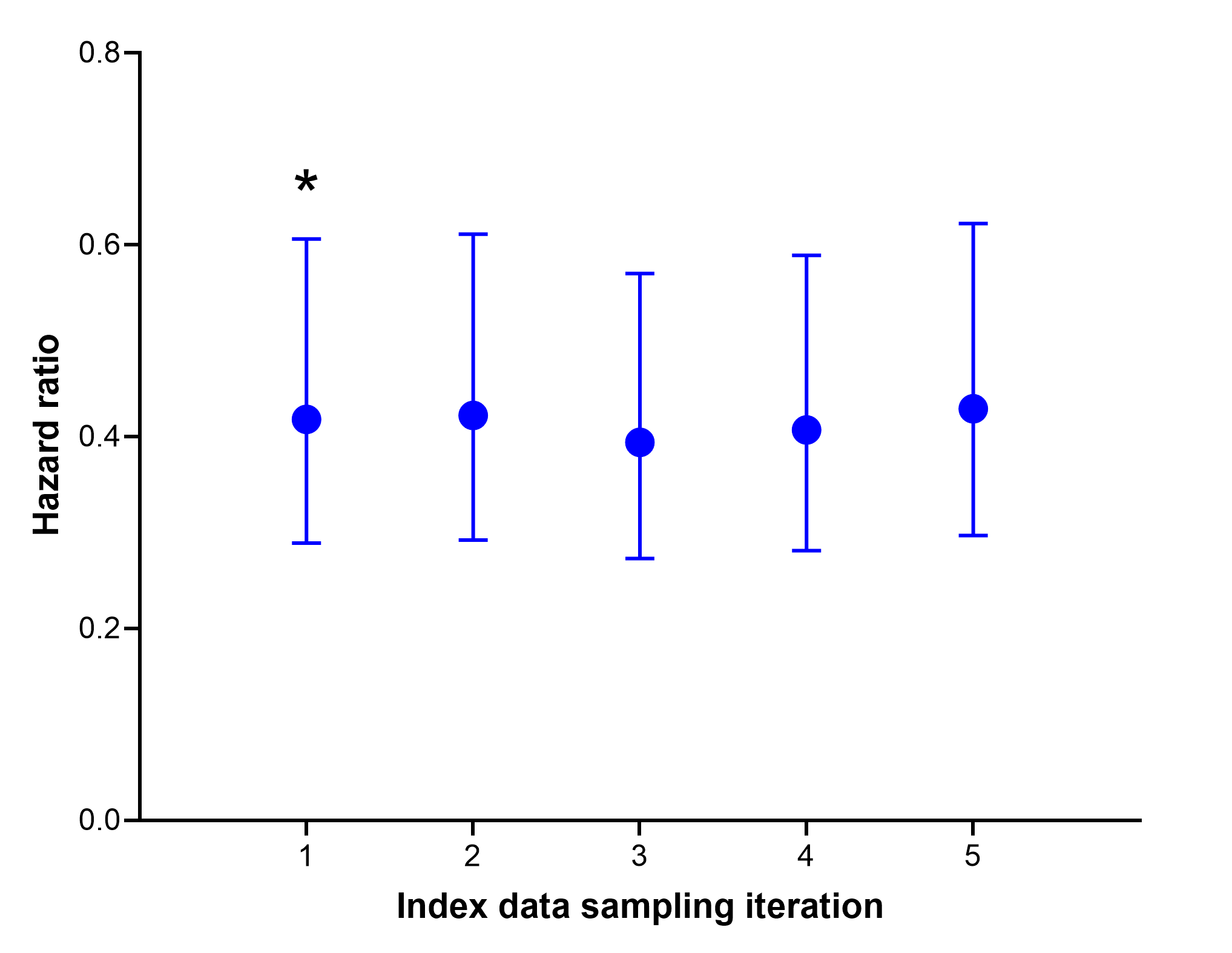


# Figure S2. Hazard ratios and 95% confidence intervals for the GLP-1 RAs group versus nonusers from fully-adjusted Cox models for DR for five iterations of index date random sampling.


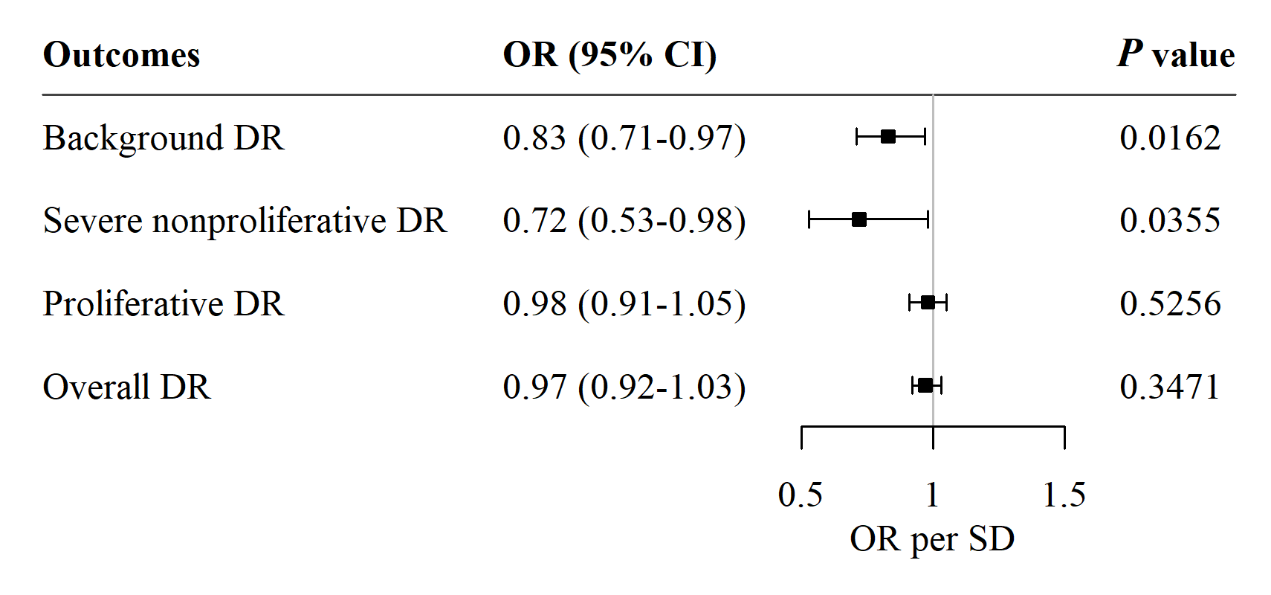


# Figure S3. The association of a 1-SD increase of GLP1R gene expression in pancreas tissue with the risk of DR using SMR method.

OR, odds ratio; CI, confidence interval, DR, diabetic retinopathy.
